# The Effects of Parathyroid Hormone Applied at Different Regimes on the Trochanteric Region of the Femur in Ovariectomized Rat Model of Osteoporosis

**DOI:** 10.4061/2011/363617

**Published:** 2011-04-13

**Authors:** M. Tezval, A. Banhardt, S. Sehmisch, L. Kolios, U. Schmelz, K. M. Stuermer, E. K. Stuermer

**Affiliations:** ^1^Department of Trauma and Reconstructive Surgery, Georg-August-University of Goettingen, 37075 Goettingen, Germany; ^2^Department of Trauma and Reconstructive Surgery, University Hospital, Georg-August-University of Goettingen, Robert Koch Street No. 40, 37075 Goettingen, Germany; ^3^Medical Institute of General Hygiene and Environmental Health, Georg-August-University of Goettingen, 37075 Goettingen, Germany

## Abstract

This study aims to investigate the effects of two application frequencies of parathyroid hormone on the trochanteric region of rat femur. Forty-three-month-old female Sprague-Dawley rats were divided into 4 groups (*n* = 10/group). Three groups were ovariectomized, and 8 weeks later they were administered the following treatments (5 weeks): soy-free diet (OVX), subcutaneously injected PTH (0.040 mg/kg) 5 days a week (PTH 5x/w), subcutaneously injected PTH (0.040 mg/kg) every 2 days (PTH e2d), and a sham group. The values of the biomechanical and histomorphometric parameters showed higher results in 5x/w animals in comparison to the OVX and PTH 2ed groups. The ratio between bone diameter/marrow diameter (B.Dm/Ma.Dm) in subtrochanteric cross sections did not show any significant differences between PTH 5x/w and PTH e2d. The increased bone formation rate was observed under PTH treatment in both groups mainly at the endosteal side. The endosteum seems here to be one of the targets of PTH with an accelerate bone formation and a pronounced filling-in of intracortical cavities with higher intensity for the PTH 5x/w in comparison to PTH e2d rats.

## 1. Introduction

In the last decade, the parathyroid hormone (PTH) has become more important as a possible alternative for the treatment of postmenopausal osteoporosis. Most of the studies with PTH have focused mainly on the dose-dependent effects of this hormone or its combination with other drugs [1–4]. Since researchers found that the intermittent substitution of PTH, in contrast to its continuous application, was able to prevent postmenopausal bone loss, the question has been which application frequency would deliver the best anabolic result [5]? The time interval between the anabolic und catabolic effects of intermittent application of PTH (“anabolic window”) is the key to respond and to understand such behavior of this hormone. In addition, another unanswered question is how PTH affects different skeletal sites like the wrist, proximal tibia, vertebral bodies and, especially, proximal femur. 

It is known that the trochanteric fracture of the femur is one of the most common fracture types in menopausal women. Therefore, investigations of the strength of this skeletal site after treatment with antiosteoporotic agents seem to be important and have a high clinical relevance [6]. Such investigations, however, are rare because of the difficulty in producing a reliable trochanteric fracture in animal models [7].

The ovariectomized (OVX) rat is a well-proven animal model for osteoporosis studies [8, 9]. There are many similarities between the human and rat femur, both at the microstructural and macrostructural levels [10, 11].

In the present study, we investigated the region-specific influence of two different application frequencies of PTH on femoral trochanteric strength of ovariectomized rats.

## 2. Materials and Methods

### 2.1. Experimental Animals and Substances

The experiments were carried out using forty-three-month-old female Sprague-Dawley rats fed a standard diet ad libitum. The animals were randomized by weight into four experimental groups (*n* = 10 in each group): OVX soy-free group (OVX), PTH group receiving 5x/w), PTH group receiving subcutaneous injections of 0.040 mg/kg parathyroid hormone (1-34) every 2 days (PTH e2d), and a sham group. The experimental procedures were approved by the local ethics commission under German animal protection law (permission from 11.03.1998, AZ: 509.42502/01-02.98. Bezirkregierung Braunschweig). Eight weeks after bilateral OVX, we started the drug treatments, which were continued for the next five weeks. After five weeks of drug therapy, the rats were euthanized, and both femurs were dissected free of soft tissue to be used for biomechanical and histomorphometric tests. 

During the treatment, the animals were subcutaneously injected with four fluorescent substances (Merck, Darmstadt, Germany) to mark the process of bone formation, especially in the cortical surface [12]. The following fluorescent agents injected were xylenol orange (90 mg/kg) on day 13, calcein green (10 mg/kg) on day 18, alizarin red (30 mg/kg) on day 24/26, and tetracycline (25 mg/kg) on day 35. This agent (tetracycline) was applicated 2 hours before euthanizing the animals. It is well known that tetracyclines are able rapidly to bind to new formatted bones immediately after application.

The results of the fluorochrome labeling were analyzed in cross sections of femurs 15 mm distal from the femoral head in the subtrochanteric region [6].

### 2.2. Biomechanical Test

The biomechanical test was performed with our new breaking test as previously described [13]. In a deepening (4 mm diameter), the femoral head (left femurs) was fixed at proximal end of the breaking machine. The femoral shaft was positioned between two rotable cylinders. Force was applied with a ZWICK-testing machine, type 145660 Z020/TND (Zwick/Roell, Ulm, Germany), from the lateral side of the bone (vertically to the trochanter tertius in rat) to the greater (major) trochanter using a metallic stamp.

The range of assessment was from 2 N to 500 N. During the mechanical test, the bone had the possibility to slide between the roller clamps. The force was applied until the femur was broken in the trochanteric region. The measured curve after breaking test fulfilled the load and displacement during the test. The “slope” of our breaking curve (load-displacement-curve) corresponds to the tangent of the strength-strain-curve. This tangent demonstrates the elasticity of each material. This means that in our study the slope of the breaking curves shows the elasticity of the bones (femurs). Load and displacement were recorded, and ultimate maximal breaking strength (maximal load, F_max_ (N)) and stiffness (elasticity, slope of the linear part of the curve, N/mm) were calculated. 

The measurement of yield point (yield load) of bone is not easy. In the opinion of many researches, it is only possible to measure the “yield area” and not “a point.” The mean reason for this is the inhomogeneity of bone (consist of mineral, organic material and many other elements and water). 

Using the curve of F(max) (maximal load) and the load displacement, we can only measure the yield area. 

The work of Brzo'ska et al. confirmed the fact that after breaking the femoral neck they could see different curves. In their study, this fact made the measurement of yield load of femoral neck more difficult. Our previous experiments could, however, show that the yield area also in our femurs corresponds approximately to both standard deviations.

We defined the yield point (load) as a decrease in elasticity (stiffness) of more than twice the standard deviation (SD) [14].

### 2.3. Radiography of Fractures

**Figure 1:**
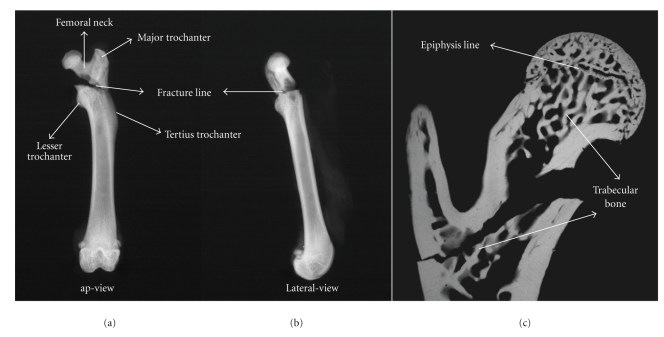
Radiographs of proximal rat femurs after breaking test. (a) Anterior-posterior view (ap-view) of the reversed trochanteric fracture of rat femur (type A3 fracture according to AO classification). (b) Lateral view. (c) The figure shows the microarchitecture (microradiograph of sagittal section) of proximal femur of Sprague-Dawley rat. Please note the fracture line and the content of the trabecular bone in this area.

X-ray radiography (in the anterior-posterior and lateral view) of all left femurs was performed in the study (Figures 1(a) and 1(b)). For this, we used a special Kodak-film (Kodak SR type 45) and a Faxitron fine-focus cabinet X-ray system (model 43855A; Faxitron X-ray System) with 40 kV.

### 2.4. Cancellous and Cortical Bone Histomorphometry

The left femurs were fixed, after the biomechanical test, in 70% ethanol for 2 days (48 hours), dehydrated through an alcohol gradient, and at least embedded in methyl methacrylate gel. Sagittal, sections (150 *μ*m thick) of the embedded proximal femur were prepared using a microtome (Leica, Sawmicrotom 1600). The target region of embedded femur for the histomorphometry analysis was the frame between the epiphyseal zone and the femoral intertrochanteric line (2 mm distally). These microradiographs of the femoral sections were used to analyze the histomorphometric changes in the trabecular surfaces (Figure 1(c)). 

We used a digitizing morphometric system to measure and analyze bone histomorphometric parameters. The system consisted of a microscope (Leica-System MZ 7.5), a digitizing pad coupled to a personal computer with an additional morphometry program (Qwin software). 

We measured trabecular area (Tb.Ar), the number of trabecular nodes (N.Nd), trabecular connectivity (N.Nd/mm^2^), and mean trabecular width (Tb.Wi) [15]. 

A problem is the very difficult measurement of the cortical changes at cortical surface in medial proximal femoral neck. The proximal (medial) part of the femoral neck in rats and other large animals seems not to be covered by periosteal tissue. This is an important factor to consider, especially when anabolic agents are tested with pronounced periosteal stimulation. In contrast, the trochanteric region contains a cortical surface covered by a sufficient periosteum. Furthermore, the trochanteric region has a high content of trabecular net.

Because measurable changes in the cortical area occur first long time after OVX, the real early changes of thickness in this region remain difficult to measure. 

**Figure 2:**
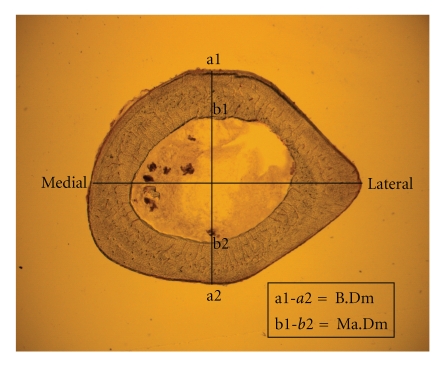
Cortical bone analysis. The figure shows the trochanteric cross section of proximal femur of Sprague-Dawley rat, cut 15 mm distal from the capitis femoris. We measured the bone diameter (B.DM) and the marrow diameter (Ma.Dm) on the ventrodorsal axis (the line perpendicular to the middle of the mediolateral axis) of the cross section.

We measured the ratio between diameter of femoral bone (B.Dm) and marrow diameter (Ma.Dm) in the cross sections 15 mm distal of the capitis femoris (femoral head) in the subtrochanteric area [6]. We assessed the B.Dm of the cross sections in the midline of the cross sections (dorso-ventral-axis) and on the same line as the Ma.Dm as previously described (Figure 2). [6] It is here important to mention that this area in rat is a region between major trochanter, minor trochanter, and tertius trochanter. This means that this part (in rat) belongs to “the trochanteric region”. The further advantage of using the proximal femur (15 mm distal to the femoral head) is the opportunity to have a relatively homogenous width of cortical surface and its independence to the technical problems during providing sufficient cross sections.

### 2.5. Serum Analysis for Bone Anabolic Markers

Blood samples (5 mL) were collected from the sacrificed animals and centrifuged at 3000 g for 10 minutes. The serum was stored at −20°C until the electrochemiluminescence immunoassay (ECLIA, Roche diagnostics, Mannheim, Germany) was performed. The level of osteocalcin was measured in the serum. A further marker of bone remodeling, alkaline phosphatase (AP), was also quantitatively determined.

### 2.6. Ashing

To determine the amount of mineralized bone, the right femurs were mineralized at 750°C and weighed to the nearest 10^−5^ g. The femurs were weighed (dry) before and after ashing. At the end of the experiment, the mineral content of each bone was indicated as a percentage of the total weight of the same femur (weight after ashing/weight before ashing).

### 2.7. Statistics

The mean values of the differences between the study groups in all of the comparative bioassays were assessed using one-way ANOVA test with Tuckey_kramer post hoc test. (Prism TM 4.0, Graph Pad, San Diego, USA). *P* values <.05 were considered significant.

## 3. Results

### 3.1. Body Weight and Mineral Content

As demonstrated in Table 1, there were no significant differences in body weight between the groups at the beginning of the study. At the end of experiment, we observed a significant weight gain in all groups compared to the sham group (Table 1).

After ashing of the left femurs, both PTH 5x/w and PTH e2d groups (49.54% and 48.30%, resp.) showed significantly higher mineral content and similar mineral content than that of the sham group compared to the OVX (45.80%) group. Although the mean value in the PTH 5x/w rats was higher than the PTH e2d animals, the difference in mineral content was not statistically significant. In addition, the OVX animals clearly had less mineral content in comparison to the sham (49.68%) rats.

### 3.2. Biomechanical Test

The mean values of maximal load (F_max_), stiffness, and yield load (yL) showed higher results after treatment with parathyroid hormone 5x/w (F_max_ = 194.1 N, stiffness = 347.6 N/mm, yL = 134.6 N) in comparison to the e2d group (F_max_ = 176.3 N, stiffness = 250.9 N/mm, yL = 110.3 N). These results were statistically significant for stiffness. Concerning biomechanical parameters, the results of the PTH 5x/w animals showed a significant improvement compared to the OVX rats, but there were no significant differences between the OVX group (F_max_ = 169.3 N, stiffness = 230.2 N/mm, yL = 86.96 N) and the PTH e2d group. 

Concerning stiffness and yield load, the sham animals had higher results (F_max_ = 187.0 N, stiffness = 294.8 N/mm, yL = 120.6 N) than OVX rats (Table 1). Concerning biomechanical parameters, the results of the PTH 5x/w animals showed a significant improvement compared to the OVX rats, but there were no significant differences between the OVX group (F_max_ = 169.3 N, stiffness = 230.2 N/mm, yL = 86.96 N) and the PTH e2d group. The mean values of maximal load (F_max_), stiffness, and yield load (yL) showed higher results after treatment with parathyroid hormone 5x/w (F_max_ = 194.1 N, stiffness = 347.6 N/mm, yL = 134.6 N) in comparison to the e2d group (F_max_ = 176.3 N, stiffness = 250.9 N/mm, yL = 110.3 N). These results were statistically significant for stiffness. 

### 3.3. Serum Analysis

Serum osteocalcin levels differed between both PTH-treated groups (*P* < .05) in comparison to the sham and OVX animals. There were no significant differences in serum OC levels between the PTH 5x/w (37.43 ng/mL) and PTH e2d groups (32.55 ng/mL). In sham animals (14.59 ng/mL), OC levels were lower compared to OVX rats (17.83 ng/mL), but the results were not significant. The concentration of AP in the PTH e2d group (74.27 ng/mL) was increased, but this result was only significant compared to the sham group (40.90 ng/mL) (Table 1).

### 3.4. Histomorphometry Analysis

Table 2 shows the results of histomorphometric tests. 

The number of trabecular nodes/mm^2^ (N.Nd/mm^2^ = connectivity) was significantly higher in sham (21.34) and PTH e2d (19.10) groups compared to OVX rats (11.91). Conversely, there were no significant differences in connectivity between the PTH 5x/w and PTH e2d groups. The PTH 5x/w rats demonstrated better results concerning Tb.Ar and Tb.Wi in comparison to PTH e2d animals. However, the only statistically significant difference between the PTH groups was for Tb.Wi. Both PTH-treated groups (5x/w and e2d) presented improved results for Tb.Ar (81.54% versus 73.38%) and Tb.Wi (17.59 versus 14.65 *μ*m) compared to OVX rats (Tb.Ar = 41.15%, Tb.Wi = 11.51 *μ*m). Concerning Tb.Ar and Tb.Wi, the PTH 5x/w showed significantly better results than the sham group (Tb.Ar = 66.85%, Tb.Wi = 12.24 *μ*m).

To determine the changes in the cortical surface of the femurs, we measured the diameters of subtrochanteric bone cross sections (B.Dm) and the marrows (Ma.Dm) in the ventro-dorsal axis in all groups. The B.Dm/Ma.Dm ratio was able to compare even minimal changes in the cortical width of the subtrochanteric region of the rat femur among all groups. Although we did not see any significant changes between any of the groups concerning B.Dm, the mean values of Ma.Dm were significantly lower in both PTH-treated groups compared to OVX animals. The mean values of the B.Dm/Ma.Dm ratio were significantly higher in both PTH 5x/w rats (1.843) and PTH e2d rats (1.805) compared to the OVX group (1.652). The PTH 5x/w rats also showed a significantly higher B.Dm/Ma.Dm ratio compared to the sham animals (1.726) (Table 2).

**Figure 3:**
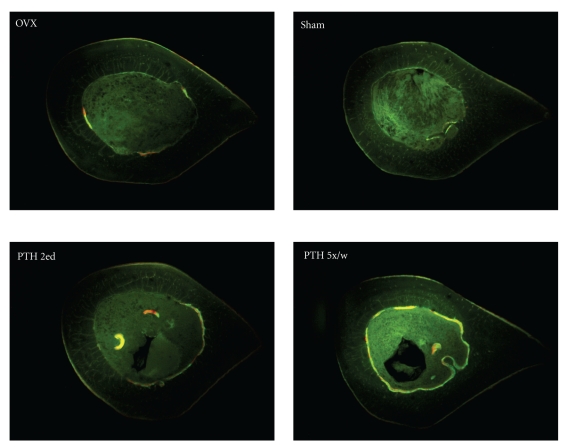
Analysis of fluorescence apposition bands in transversal sections from the subtrochanteric region of rat femur. The sections (all sections 15 mm distal from femoral head) were studied by fluorescence microscopy. In the OVX group, we could mainly observe a periosteal activity. In the sham group, only a minimal periosteal bone formation could be seen. The PTH e2d-treated animals showed weaker endosteal appositions in comparison to the PTH 5x/w animals.

These results in addition to the results of fluorescence microscopy could show useful information about endosteal and periosteal bone remodeling (apposition bands) within the cortical surface. The increased bone formation rate was observed under PTH treatment in both groups mainly at the endosteal side by fluorescent microscopic analysis of the cross sections from the proximal femur. The endosteum seems here to be one of the targets of PTH with an accelerate bone formation and a pronounced filling-in of intracortical cavities with higher intensity for the PTH 5x/w in comparison to PTH e2d rats (Figure 3).

## 4. Discussion

The paradoxical effects of PTH on bone were first described by Selye in 1932. He observed that continuous intravenous administration of PTH was able to elevate predominantly the bone resorption [6]. In contrast, the intermittent administration of PTH mainly resulted in a stimulation of bone formation, especially in the trabecular area [16]. In the last several years, studies have emphasized the importance of evaluating the effects of this hormone in cortical areas [16, 17]. 

Although many studies could confirm the anabolic effect of PTH on bone, the question of which application frequencies produce the best results is still controversial. Another important question is in which skeletal sites (proximal tibia, vertebral bodies, etc.) would we expect to see detectable positive antiosteoporotic effects of this hormone [18]. The trochanteric region of rat femur contains major trochanter, minor trochanter, and tertius trochanter. The area between these three trochanters was the region of interest in our studies. The femur is one of the most important skeletal sites in postmenopausal osteoporosis. Next to the femoral neck fracture, the trochanteric fracture of the femur is one of the most common fracture types in elderly women [6]. This type of fracture presents a surgical challenge as well as an economical problem. This part of the rat femur contains both trabecular and cortical bone, in contrast to the femoral shaft [9]. Therefore, the intertrochanteric part of the femur seems to be an important region to investigate the biomechanical changes after therapy with antiosteoporotic substances like the parathyroid hormone because PTH appears to influence both cortical and trabecular bone surfaces [6, 19]. Hence, it is important to look for therapy options and drugs that prevent such fractures in postmenopausal osteoporotic bone.

The intermittent administration of PTH gained more importance in the last decade as a possible alternative for the treatment of osteoporosis and prophylaxis of fractures. In the present study, we investigated two therapy options with PTH. We compared the anabolic effect of PTH on the proximal femur of OVX rats after five weeks of subcutaneous injections (0.040/kg), five days a week, or subcutaneous injections every 2 days.

At the end of experiment, we observed a significant weight gain in OVX group compared to the sham group. Neither the PTH 5x/w nor the PTH e2d treatment could prevent the weight gain caused by OVX. Indeed, the sham animals had the lowest weight gain of all of the rats.

When assessed by the breaking test, femurs of the PTH 5x/w treatment reached the strength level of sham rats. Although the treatment with PTH e2d also led to better mean values in the biomechanical test, the results were not statistically significant in comparison to OVX rats. The changes observed in the biomechanical test were partially evident when bones from PTH 5x/w- and PTH e2d-treated rats were examined by histomorphometry.

In our opinion, the main reason for significantly better results of Tb.Ar in PTH 5x/w rats was the improvement of trabecular thickness in these animals. The age-dependent study of Fridle et al., however, showed an increase in trabecular number in younger rats, whereas older rats demonstrated increases in trabecular thickness [3]. Another study also showed similar age-related effects of PTH [20].

Therapy with bone anabolic agents causes serum levels of the bone formation markers OC and AP to increase, and we observed similar results after PTH treatment. However, we did not observe any statistically significant differences between the two PTH application paradigms in our study. In case of AP, this could be due to the little sensitivity and specificity of this bone formation marker.

Some studies have shown that after PTH treatment, the addition of bone to the periosteal surface seems to be responsible for a much greater contribution to bone strength than bone added to the endosteal surface [19]. In contrast, the present work demonstrated increases in the B.Dm/Ma.Dm ratio in both PTH-treated animals. This finding was caused by a decrease in Ma.Dm rather than an increase in B.Dm. In our study, the fluorescence apposition bands in the endosteal side of femoral cross sections after PTH treatments underline this effect. Although the B.Dm/Ma.Dm-ratio was in the PTH 5x/w higher than PTH e2d rats, the results were not statistically significant. But in our opinion, the higher dosages of PTH seem to have more intensive anabolic effect on the endosteal side of the cortical surface. Additional experiments, however, are necessary to underline such effect. It remains unclear, however, if the dose of PTH plays any role. Komatsu et al. also showed that PTH induced new bone formation at endocortical (endosteal) surfaces [1]. Interestingly, in a fracture healing rat model, the same authors found that PTH dose dependently (30 *μ*g/kg) stimulated bone formation within the intramedullary cavity [1]. 

In the ash test, higher total doses of 1–34 PTH induce a better but not statistically significant improvement on BMC, reversing the effects that the OVX has on this quantitative determinant of bone strength. 

Many of the measured parameters in our work did not show any statistically significant differences between the PTH 5x/w and PTH e2d groups, but the mean values in most of the tests were slightly higher in the PTH 5x/w animals compared to the PTH e2d animals. In our opinion, the antiosteoporotic effects of PTH 5x/w seem to be slightly stronger than PTH e2d treatment although we saw only in Tb.Wi and in stiffness statistically significant differences. 

The exact signaling pathways of the anabolic effect of PTH are not clear, but the pathways activated after PTH treatment determine whether this hormone has catabolic or anabolic actions [21]. The parathyroid hormone (PTH) stimulates the processes that lead to bone formation prior to stimulating the pathways associated with bone resorption. The “window” between both of these effects is not clear [22]. Rubin and Bilezikian showed that the bone formation markers reached a maximum level within a few days after PTH treatment, whereas the bone resorption parameters had the maximum level after approximately 3 weeks [21, 22]. We believe that during these three weeks between the maximal anabolic and maximal catabolic phases of PTH, named “anabolic window” by Rubin and Bilezikian, different PTH application frequencies could lead to an improvement of bone strength; however, this would probably occur with different intensities. It is important to mention that the half-life of PTH after a single application as well as the dose and skeletal site where the anabolic effect of PTH is investigated are important aspects that play critical roles in determining the effects of PTH. Therefore, future investigations should evaluate the time- and dose-dependent changes of the trochanteric region after PTH therapy. Furthermore, additional experiments should be performed in animals of different ages and in male rats.

## 5. Conclusions

The trochanteric region of the rat femur is an important skeletal site for osteoporosis studies because of its high clinical relevance. The present study showed that treatment with PTH 5x/w and PTH 2ed both improved the biomechanical and histomorphometric properties and partially reversed the effects of OVX in the trochanteric region of the rat femur. Under conditions presented in our study, the anabolic effect of PTH 5x/w seems to be slightly stronger than PTH e2d therapy, but the main significant changes were observed concerning elasticity and Tb.wi. Further experiments are needed to determine which form of PTH therapy options is able to effectively prevent trochanteric fractures. Thus, future studies related to dose- and time-related investigations should be conducted.

## Figures and Tables

**Table 1:** Study results. Body weight, mineral content, biomechanical test, and serum analysis of anabolic bone parameters.

	SHAM		OVX		PTH 5x/w		PTH e2d	
	Mean	STD	Mean	STD	Mean	STD	Mean	STD
Body weight								
Before OVX (g)	247.1	8.17	236.7	19.71	240.0	14.67	246.6	10.41
At the end of trial (g)	274.3^a^	15.91	341.0	23.64	341.4^b^	24.95	346.2^b^	32.48

Mineral content (after ashing) in left femurs (%)	49.68^a^	1.93	45.80^b^	1.68	49.54^a^	1.89	48.30^a^	1.41

Biomechanical test								
F_max_ (N)	187.0	20.81	169.3	25.38	194.1^a^	22.78	176.3	17.97
Stiffness (N/mm)	294.8^a^	68.19	230.2^b^	60.62	347.6^ac^	39.78	250.9	20.73
Yield load (N)	120.6^a^	28.67	86.96^b^	26.53	134.6^a^	25.77	110.3	9.6

Serum analysis								
Osteocalcin (OC) (ng/mL)	14.59	7.51	17.83	6.67	32.55^ab^	6.26	37.43^ab^	9.63
Alkaline Phosphatase (AP)	40.90	21.62	59.83	21.62	59.54	15.04	74.27^b^	21.99

OVX (Ovariectomy), F_max_ (maximal load).

The *P* value of the difference between treated and untreated animals was calculated using a one-way ANOVA. *P* values <.05 were considered significant.

^a^ *P* < .05 versus OVX, ^b^ *P* < .05 versus sham, and ^c^ *P* < .05 PTH 5x/w versus PTH e2d.

**Table 2:** Results of the histomorphometry analysis.

	SHAM		OVX		PTH 5x/w		PTH e2d	
	Mean	STD	Mean	STD	Mean	STD	Mean	STD
Histomorphometry								
Connectivity (N.Nd/mm^2^)	21.34^a^	5.52	11.91^b^	5.69	15.03^b^	2.86	19.10^a^	1.34
Trabecular area (Tb.Ar) (%)	66.85^a^	10.19	41.15^b^	9.5	81.54^ab^	8.96	73.38^a^	8.73
Trabecular width (Tb.Wi) (*μ*m)	12.24	1.54	11.51	1.31	17.59^abc^	3.25	14.65^a^	1.31
Trabecular nodes (N.Nd)	58.33	15.56	37.60	26.56	42.90	12.5	54.57	11.65

Histomorphometry cortical subtrochanter								
Bone diameter (B.Dm) (*μ*m)	3228	203.6	3188	104.3	3217	75.42	3231	114.6
Marrow diameter (Ma.Dm) (*μ*m)	1872	151.2	1933	102.4	1747^a^	87.39	1795^a^	99.61
Ratio (B.Dm/Ma.Dm)	1.726	0.073	1.652	0.058	1.843^ab^	0.068	1.805^a^	0.133

The *P* value of the difference between treated and untreated animals was calculated using a one-way ANOVA. *P* values <.05 were considered significant.

^a^ *P* < .05 versus OVX, ^b^ *P* < .05 versus sham, and ^c^ *P* < .05 PTH 5x/w versus PTH e2d.
